# The impact of online classes on sleep, physical activity, and cognition functioning among physical education students

**DOI:** 10.3389/fpsyg.2024.1397588

**Published:** 2024-05-30

**Authors:** Monoem Haddad, Zied Abbes, Abdel-Salam G. Abdel-Salam

**Affiliations:** ^1^Physical Education Department, College of Education, Qatar University, Doha, Qatar; ^2^Department of Mathematics and Statistics, College of Arts and Sciences, Qatar University, Doha, Qatar

**Keywords:** wrist actigraphy, cognitive assessment, learning environment, step counter, university students

## Abstract

**Introduction:**

Online education has become a crucial component of teachers’ professional development, and universities incorporate innovative pedagogical approaches to enhance teachers’ training. These approaches have proven invaluable, particularly during the COVID-19 pandemic. This study investigates the impact of online versus face-to-face learning environments on sleep quality, physical activity, and cognitive functioning among physical education students.

**Methods:**

Utilizing a unique methodological approach that combines wrist actigraphy, the Pittsburgh Sleep Quality Index, and the Cambridge Neuropsychological Test Automated Battery, we provide a comprehensive assessment of these variables. Over 4 weeks, 19 male students participated in alternating online and face-to-face class formats.

**Results:**

Our results reveal no significant differences in sleep quality or cognitive function between learning environments. However, notable findings include significant differences in Paired Associates Learning and weekday step counts in the face-to-face setting.

**Discussion:**

These insights suggest that while online learning environments may not adversely affect sleep or cognitive functions, they could impact certain aspects of physical activity and specific cognitive tasks. These findings contribute to the nuanced understanding of online learning’s implications and can inform the design of educational strategies that promote student well-being.

## 1 Introduction

Online education has become an essential element of teachers’ professional development, and universities are responsible for enhancing their training by incorporating innovative pedagogical approaches that have proven invaluable under various circumstances, including the COVID-19 pandemic. This study was conducted at Qatar University, located in Doha, Qatar, providing a unique context for exploring the impacts of online versus face-to-face learning environments within the Arab region. Prioritizing the creation of engaging learning opportunities and cultivating unique educational settings using digital technologies has become a central focus of the higher education system. This approach aims to enable teachers and students with limited exposure to e-learning to adapt swiftly to current challenges (Rapanta et al., 2020).

The challenges brought about by changes enforced by the pandemic in the realm of teaching activities were multifaceted. They encompass issues such as heightened anxiety, diminished face-to-face interactions, shifts from conventional teaching techniques, the demand for indoor physical activities with associated issues, and increased workload and stress stemming from novel working conditions (Aperribai et al., 2020). Additionally, Joshi et al. (2021) emphasize the unique challenges within the academic landscape in India that impede the enhancement of quality education in universities. These obstacles include limited funding for the procurement of advanced information technology (IT) equipment, inadequate computer skills training, external distractions and family responsibilities, technical issues, instructional and assessment difficulties, a pessimistic outlook, and lack of motivation. Given the convenience of attending online classes from home, it is important to note that this method of teaching often involves prolonged exposure to screens on mobile devices, tablets, or laptops. Consequently, students may need to remain sedentary for extended periods. Prolonged exposure to artificial light from electronic devices can adversely affect human health (Khare et al., 2021). In addition to these challenges, the increasing prevalence of nomophobia and problematic internet use among students has raised concerns regarding their potential impact on learning environments. Nomophobia, or the fear of being without a mobile phone, has been linked to heightened anxiety and could interfere with students’ learning processes and overall well-being. Relevant studies suggest a high prevalence of nomophobia among university students (Tuco et al., 2023). In Arab countries, moderate to severe rates of nomophobia have been observed, indicating its potential influence on students’ academic engagement and performance (Jelleli et al., 2023). Similarly, problematic internet use, characterized by excessive and uncontrolled online activity, has been associated with adverse mental health outcomes and could affect students’ ability to engage effectively in online learning. A meta-analytic review reported high rates of problematic internet use and its associations with mental health outcomes among students (Cai et al., 2023). Additionally, the prevalence and risk factors of internet gaming disorder and problematic internet use have garnered attention, especially during the COVID-19 pandemic, underscoring the need to understand these behaviors in the context of online education (Oka et al., 2021). Given the significant role of sleep in determining cognitive and physical performance, its inclusion as a study variable is essential. Sleep quality and duration have profound effects on learning efficiency, memory consolidation, and overall student health, which are particularly pertinent in the context of varied learning environments such as online versus face-to-face settings (Curcio et al., 2006; Walker and Stickgold, 2006). These considerations support our focus on assessing how different learning modalities influence sleep patterns among physical education students (Alhola and Polo-Kantola, 2007; Killgore, 2010). To address the unique impacts of the pandemic, our study contrasts online and face-to-face learning environments during this period, specifically incorporating an analysis of stress and anxiety as they relate to these educational settings. This consideration aims to enhance our understanding of how pandemic-related factors influence sleep, memory, and cognitive functions among physical education students. Our study specifically examines both the theoretical and practical aspects of physical education, integrating components of physical training and theoretical instruction within both the online and face-to-face learning environments. This comprehensive approach allows us to evaluate the impact of these modalities on various aspects of student well-being, including sleep, physical activity, and cognitive functions. Tan et al. (2020) suggest that the university environment can view the pandemic as an opportunity for innovation and exploration of novel teaching methods in online distance learning.

Challenges in online education have been documented in Ghana, where students face difficulties owing to the limited computer and technical proficiency required for remote learning. In addition, parents struggle to assist their children in accessing and navigating online platforms, and Internet connectivity is often constrained. Consequently, the pandemic has adversely affected the quality of education and learning experiences. It is imperative to provide training to both students and teachers to ensure the efficient utilization of these online platforms (Owusu-Fordjour et al., 2020). Over time, significant advancements have been made in the field of online teaching and learning. Despite the intricacies in transitioning to a fundamentally different teaching approach, our university serves as an exemplary institution that has successfully risen to the challenge of finding solutions to ensure the uninterrupted continuity of education during the pandemic (Heider, 2021).

The impact of the pandemic on university staff and students has primarily manifested as reduced physical activity due to the suspension of in-person teaching and the closure of fitness facilities. However, Barkley et al. (2020) indicate that this decline was more pronounced among those with high levels of physical activity. Conversely, individuals with medium and low levels of physical activity expressed increased concern about maintaining their physical activity routines. By contrast, López-Valenciano et al. (2021) observe a decrease in engagement in lower-intensity physical activities such as walking and very intense/vigorous physical activity among students from various countries. Nevertheless, they exhibited a consistent commitment to maintain a minimum level of physical activity during the pandemic, especially if they were already active before the outbreak. Meza and López (2021) also report a decrease in overall physical activity engagement, but noted that some individuals were compensated by organizing physical activities at home. Concerning PA-related issues, Rodríguez-Larrad et al. (2021) conduct a study on 13,754 Spanish students from 16 universities. They find a decrease in both moderate and vigorous physical activity levels, along with an increase in sedentary behavior in over 50% of the cases. However, some individuals have sought to address these deficiencies by incorporating high-intensity activities and mind–body practices, such as yoga, especially among women who excel in managing physical activities and often use social networks for this purpose. It is worth noting that during the pandemic, men took more steps than women, leading to a noticeable decline in the weekly distance covered (Wickersham et al., 2021).

The perceptions of safety measures and their impact on student lifestyles vary significantly from country to country. For example, in Denmark, a survey revealed that approximately 68% of the students adhered to government-issued protective measures. Compliance was associated with older age, feelings of depression, and challenges stemming from a pandemic. Surprisingly, despite this adherence, approximately 60% of the Danish students reported no significant concerns about the pandemic (Berg-Beckhoff et al., 2021). In Naples, Italy, the pandemic has brought about substantial changes in the lifestyles of students, with men particularly affected by negative eating habits and a decline in physical activity due to quarantine measures (Brancaccio et al., 2021). A German study finds a link between increased alcohol consumption, reduced physical activity, smoking, cannabis use, and depressive symptoms related to the pandemic among students at four universities (Busse et al., 2021). Similarly, research involving Swiss students conducted by Volken et al. (2021) reveals that more than one-fourth experienced symptoms of depression during the pandemic.

However, the impact of online teaching on sleep patterns, physical activity, and cognitive functioning among university students has not been studied extensively. Therefore, the current research aimed to explore the effects of online classes compared to traditional face-to-face classes, with a specific focus on students majoring in physical education. Our study hypothesizes that: (1) there will be no significant difference in sleep quality between online and face-to-face learning environments; (2) physical activity levels will be higher in face-to-face learning environments compared to online settings; and (3) cognitive function will remain consistent across both learning environments. These hypotheses are formulated based on the assumption that while the modality of learning might influence physical activity due to inherent differences in the physical engagement required, the impact on sleep quality and cognitive function could be less pronounced.

## 2 Materials and methods

### 2.1 Study design and participants

This study used a within-subjects design to examine the influence of distinct learning environments (face-to-face and online) on multiple facets of students’ sleep patterns, physical activity levels, and cognitive function. The study spanned 4 weeks, consisting of 2 weeks of online classes followed by 2 weeks of face-to-face classes. Nineteen male students (age: 25 ± 3 years; weight: 79.36 ± 15.18 kg; height: 1.77 ± 0.09 m; BMI: 25.09 ± 3.99) willingly took part in this research. To determine the required sample size and ensure adequate statistical power for detecting meaningful effects, *a priori* power analysis was conducted using G*Power software (Version 3.1.9.6). Based on preliminary studies and existing literature, an expected moderate effect size (Cohen’s *d*) of 0.5 was assumed for the primary outcomes. The power analysis was set with an alpha level of 0.05 and a power of 80%, which are standard values to detect true effects while controlling for Type I and Type II errors. The analysis suggested that a minimum of 17 participants would be necessary to adequately power the study. With 19 participants enrolled, our study exceeded the minimum required sample size, ensuring robustness in our findings. The study adhered to the principles outlined in the Declaration of Helsinki, and the research protocol was approved by our university’s institutional review board (QU-IRB 1467-EA/21) before the recruitment of subjects and data collection. Before participating, all participants carefully read and signed a written informed consent form. This document comprehensively explains the potential risks and benefits of the study, details the research methods and procedures, and addresses the issues related to data confidentiality. Participants were assured that they could withdraw from the study at any time without any adverse consequences.

### 2.2 Learning environments

#### 2.2.1 Face-to-face learning environment

The face-to-face learning environment consisted of interactive lectures, group discussions, and practical sessions conducted in traditional classroom settings. The instructional content encompassed theoretical concepts in physical education, supplemented by practical exercises and demonstrations to enhance students’ understanding and application of the material. Qatar University established a standardized meeting pattern to ensure the maximum use of the instructional week, to provide students with greater registration options and flexibility, and to better facilitate scheduling of instructional facilities. On Monday and Wednesday, face-to-face sessions were scheduled for 1 h and 15 min. During Sunday, Tuesday and Thursday, each face-to-face session had a duration of 50 min.

#### 2.2.2 Online learning environment

The online learning environment utilized the university’s e-learning platform to deliver lectures, multimedia presentations, and virtual simulations. Asynchronous engagement activities, including online discussions and assignment submissions, were integrated to facilitate interaction and collaboration among students. The structure and duration of online sessions mirrored that of face-to-face instruction, ensuring consistency across learning environments.

### 2.3 Measurements

Sleep measurements included three components: Wrist Actigraphy: The ActiGraph GT9X Link (Pensacola, FL, United States), which was used to assess Sleep Efficiency (%), Total Sleep Time (TST), and wake time after sleep onset (WASO). Extensive research has demonstrated the validity and reliability of ActiGraph for these measurements (Colbert et al., 2011; Sasaki et al., 2011; Hanggi et al., 2013; Anastasopoulou et al., 2014; Cellini et al., 2016).

Daily Self-Rating Scale of Sleep Quality to gauge sleep quality, a daily self-rating scale originally proposed by Hooper and Mackinnon (1995) was used. This scale allowed participants to self-report their sleep quality on a scale ranging from 1 to 7, offering flexibility in selecting fractional ratings (e.g., 2 or 3.5).

Sleep Quality Assessment (PSQI): The Pittsburgh Sleep Quality Index (PSQI) was administered during both face-to-face and online classes to assess sleep quality. The validated Arabic translation of the PSQI by Suleiman et al. (2010) was used in this study.

The Cambridge Neuropsychological Test Automated Battery (CANTAB) was used to precisely assess cognitive functioning. Widely acknowledged as the gold standard for cognitive assessment and data collection software (De Bruin et al., 2017), the CANTAB tests were conducted using a computer equipped with a touch-sensitive screen. These tests were administered and feedback was provided in a standardized and consistent manner.

Specific cognitive domains were assessed using different components of the CANTAB:

- Rapid Visual Information Processing (RVPA): This test measured sustained attention.
- Spatial Working Memory (SWMBE): SWMBE assessed executive function.
- Paired associate learning (PAL): PAL was used to evaluate visual memory and new learning abilities.

In addition to these sleep and cognitive function measurements, students’ academic performance was evaluated based on their grade point average (GPA), as indicated by their transcripts. Physical activity was estimated using a counter walking 3D pedometers. The objective indicators of physical activity were mean weekend and weekday steps. These measures provide insight into students’ sleep quality, physical activity, and other cognitive functions.

### 2.4 Statistical analysis

A Wilcoxon signed-rank test was conducted to compare the effects of the face-to-face and online learning environments on the dependent variables. This non-parametric test was chosen to evaluate the differences within the same subject across different learning environments.

Furthermore, building upon these initial findings, a linear mixed-effects model was then employed to analyze the data further, utilizing the “lme4” package in R. In this model, “Subject” was treated as a random effect to account for the repeated measures design, while GPA and Type (face-to-face vs. online) were treated as fixed effects.

A separate mixed-model was fitted to each dependent variable. These models provided estimates of fixed effects, including the intercept, GPA, and type of learning environment, along with standard errors, *t*-values, and *p*-values for each effect. The significance of the fixed effects was determined based on their *p*-values, with a threshold of *p*-value <0.05, which is typically considered statistically significant. The model outputs were used to assess the impact of the students’ GPA and the type of learning environment on each of the dependent variables, controlling for individual differences among the subjects.

## 3 Results

### 3.1 Descriptive statistics

The following table presents a comprehensive overview of various sleep- and activity-related variables across the two groups: face-to-face and online. The variables included PSQI Score, RVPA, SWMBE, PAL, Weekday Steps, Weekend Steps, sleep quality, sleep efficiency, TST, and WASO. The mean and standard deviation (SD) are provided for each variable, offering a detailed comparison between the two groups. Additionally, the table includes the combined statistics (overall) for all participants, irrespective of their group, thereby providing a holistic view of the data (Table 1).

**Table 1:** Comparison of sleep and activity-related variables between face-to-face and online groups: mean and standard deviation analysis with overall statistics.

Variable	Online		Face-to-face		Overall	
	Mean	*SD*	Mean	*SD*	Mean	*SD*
PSQI score	6.13	3.18	6.18	3.14	6.16	3.14
RVPA	0.92	0.09	0.92	0.09	0.92	0.09
SWMBE	6.89	8.56	7.87	8.29	7.38	8.38
PAL	70.39	24.25	79.50	21.90	74.95	23.40
Weekday steps	6754.38	2261.74	7505.02	2343.98	7129.70	2318.81
Weekend steps	6875.47	2317.13	6491.88	2787.15	6683.68	2553.10
Sleep quality	2.51	0.91	2.65	0.82	2.58	0.86
Sleep efficiency	94.33	2.73	94.20	2.23	94.26	2.48
TST	493.70	206.95	475.32	136.06	484.51	174.20
WASO	21.52	9.97	22.36	9.25	21.94	9.56

### 3.2 Wilcoxon signed-rank test

The following table presents the results of the Wilcoxon signed-rank tests conducted to compare various dependent variables between the face-to-face and online learning environments. These variables include sleep quality, physical activity, and academic performance. The test statistics and *p*-values for each variable are reported. This analysis aimed to determine whether there were statistically significant differences in these outcomes based on the type of learning environment with a focus on paired comparisons within the same subjects across both environments (Table 2).

**Table 2:** Wilcoxon signed-rank test results for comparing dependent variables between face-to-face and online learning environments: sleep quality, physical activity, and academic performance measures.

Variable	*V* statistic	*p*-value
PSQI score	50.5	0.605
RVPA	120	0.324
SWMBE	74	0.632
PAL	41	0.097
Weekday steps	55	0.112
Weekend steps	115	0.433
Sleep quality	46.5	0.277
Sleep efficiency	103	0.763
TST	103.5	0.748
WASO	73.5	0.398

In the analysis using Wilcoxon signed-rank tests, the findings suggested that there were no statistically significant differences between face-to-face and online learning environments across a range of dependent variables. Specifically, measures such as the PSQI Score (*V* = 50.5, *p* = 0.6051), RVPA (*V* = 120, *p* = 0.3242), and SWMBE (*V* = 74, *p* = 0.6315) demonstrated *p*-values significantly above the conventional significance threshold of 0.05, indicating no meaningful differences in these aspects between the two learning modalities. Similarly, variables related to physical activity and sleep, including PAL (*V* = 41, *p*-value = 0.09741), weekday and weekend steps (*V* = 55, *p* = 0.1119 and *V* = 115, *p*-value = 0.4326, respectively), sleep quality (*V* = 46.5, *p*-value = 0.2774), sleep efficiency (*V* = 103, *p*-value = 0.7628), total sleep time (TST; *V* = 103.5, *p*-value = 0.7475), and wake time after sleep onset (WASO; *V* = 73.5, *p*-value = 0.3978), all yielded *p*-values that did not reach statistical significance. These results suggest that there were no substantial differences in these variables between students participating in the face-to-face and online learning settings.

### 3.3 Linear mixed-effects model

This study was designed to investigate how different learning environments, specifically face-to-face and online settings, affect students’ sleep patterns and physical activity. This investigation employed linear mixed-effects models to evaluate the influence of learning environment type on an array of variables, including sleep quality, physical activity, and other related factors. Additionally, the students’ GPA was incorporated as an independent covariate to account for academic performance, which may interact with their sleep and activity patterns (Table 3).

**Table 3:** Linear mixed-effects model analysis with GPA as covariate.

Dependent variable	Fixed effect	Estimate	*SE*	df	*t* value	*p*-value
PSQI Score	Intercept	4.981	5.657	17.032	0.881	0.391
	Type (Online)	0.053	0.346	56	0.152	0.880
	GPA	0.354	1.728	17	0.205	0.840
RVPA	Intercept	0.943	0.126	17.138	7.507	<0.001
	Type (Online)	−0.004	0.016	56	−0.248	0.805
	GPA	−0.006	0.038	17	−0.160	0.874
SWMBE	Intercept	10.801	11.541	17.163	0.936	0.362
	Type (Online)	0.974	1.594	56	0.611	0.544
	GPA	−1.203	3.519	17	−0.342	0.737
PAL	Intercept	126.519	27.619	17.226	4.581	<0.001
	Type (Online)	9.105	4.487	56	2.029	0.047
	GPA	−17.280	8.413	17	−2.054	0.056
Weekday steps	Intercept	5065.490	3550.890	17.09	1.427	0.172
	Type (Online)	750.630	374.150	56	2.006	0.050
	GPA	520.000	1083.670	17	0.480	0.638
Weekend steps	Intercept	4566.320	3093.060	17.25	1.476	0.158
	Type (Online)	−383.590	529.630	56	−0.724	0.472
	GPA	710.970	941.790	17	0.755	0.461
Sleep quality	Intercept	3.044	1.458	17.055	2.088	0.052
	Type (Online)	0.139	0.117	56	1.186	0.241
	GPA	−0.165	0.445	17	−0.371	0.716
Sleep efficiency	Intercept	92.018	2.989	17.257	30.786	<0.001
	Type (Online)	−0.132	0.517	56	−0.254	0.800
	GPA	0.712	0.910	17	0.782	0.445
TST	Intercept	937.270	224.930	17.17	4.167	0.001
	Type (Online)	−18.380	31.900	56	−0.576	0.567
	GPA	−136.570	68.570	17	−1.992	0.063
WASO	Intercept	26.819	14.928	17.092	1.797	0.090
	Type (Online)	0.842	1.553	56	0.542	0.590
	GPA	−1.631	4.556	17	−0.358	0.725

### 3.4 Pittsburgh sleep quality index (PSQI) score

In the PSQI Score analysis, the results indicated no significant effects related to the type of learning environment (online), with an estimated effect of 0.053 (*SE* = 0.346, *t* = 0.152, *p* = 0.880). Furthermore, the intercept was not statistically significant (estimate = 4.981, *SE* = 5.657, *t* = 0.881, *p* = 0.391), suggesting no substantial baseline differences in sleep quality. The GPA factor did not significantly influence the PSQI Score (estimate = 0.354, *SE* = 1.728, *t* = 0.205, *p* = 0.840).

### 3.5 RVPA

For the RVPA, there was no significant variation between the learning environments (Online: Estimate = −0.004, *SE* = 0.016, *t* = −0.248, *p*-value = 0.805). However, the intercept was statistically significant (estimate = 0.943, *SE* = 0.126, *t* = 7.507, *p*-value <0.001), indicating a notable baseline RVPA score. As a covariate, GPA was not found to significantly impact RVPA (estimate = −0.006, *SE* = 0.038, *t* = −0.160, *p* = 0.874).

### 3.6 SWMBE

In the SWMBE analysis, the type of learning environment (online) did not present significant differences (estimate = 0.974, *SE* = 1.594, *t* = 0.611, *p* = 0.544), nor did the intercept show any statistical significance (estimate = 10.801, *SE* = 11.541, *t* = 0.936, *p* = 0.362). Similarly, GPA did not significantly affect SWMBE scores (Estimate = −1.203, *SE* = 3.519, *t* = −0.342, *p*-value = 0.737).

### 3.7 PALTEA (PAL) %

Regarding PAL, a significant difference was observed between learning environments (Online: Estimate = 9.105, *SE* = 4.487, *t* = 2.029, *p* = 0.047), and the intercept also showed statistical significance (estimate = 126.519, *SE* = 27.619, *t* = 4.581, *p* < 0.001). This suggests a baseline level and an effect of online learning on PAL. GPA was found to have a nearly significant influence on PAL (Estimate = −17.280, *SE* = 8.413, *t* = −2.054, *p*-value = 0.056).

### 3.8 Mean weekday and weekend steps

The analysis of mean weekday steps revealed a significant effect of learning environment type (Online: Estimate = 750.630, *SE* = 374.150, *t* = 2.006, *p*-value = 0.050), but the intercept was not significant (estimate = 5065.490, *SE* = 3550.890, *t* = 1.427, *p*-value = 0.172). GPA did not show a significant effect (Estimate = 520.000, *SE* = 1083.670, *t* = 0.480, *p* = 0.638). For weekend steps, neither the type of learning environment (Online: Estimate = −383.590, *SE* = 529.630, *t* = −0.724, *p*-value = 0.472) nor GPA (Estimate = 710.970, *SE* = 941.790, *t* = 0.755, *p*-value = 0.461) showed significant effects, and the intercept was also not significant (Estimate = 4566.320, *SE* = 3093.060, *t* = 1.476, *p*-value = 0.158).

### 3.9 Self-rating scale of sleep quality

The self-rating scale of sleep quality did not exhibit significant differences for the type of learning environment (Online: Estimate = 0.139, *SE* = 0.117, *t* = 1.186, *p*-value = 0.241) or GPA (Estimate = −0.165, *SE* = 0.445, *t* = −0.371, *p*-value = 0.716). However, the intercept was nearly significant (estimate = 3.044, *SE* = 1.458, *t* = 2.088, *p*-value = 0.052), suggesting a trend toward a baseline effect on sleep quality ratings.

### 3.10 Sleep efficiency %

In terms of sleep efficiency, no significant effects were observed for the type of learning environment (Online: Estimate = −0.132, *SE* = 0.517, *t* = −0.254, *p*-value = 0.800) or GPA (Estimate = 0.712, *SE* = 0.910, *t* = 0.782, *p*-value = 0.445). However, the intercept was highly significant (estimate = 92.018, *SE* = 2.989, *t* = 30.786, *p*-value <0.001), indicating pronounced baseline sleep efficiency.

### 3.11 Total sleep time (TST) and wake time after sleep onset (WASO)

For TST, no significant differences were found between the learning environments (Online: Estimate = −18.380, *SE* = 31.900, *t* = −0.576, *p*-value = 0.567) or due to GPA (Estimate = −136.570, *SE* = 68.570, *t* = −1.992, *p*-value = 0.063), although the intercept was significant (Estimate = 937.270, *SE* = 224.930, *t* = 4.167, *p*-value <0.001). In the case of WASO, neither the type of learning environment (Online: Estimate = 0.842, *SE* = 1.553, *t* = 0.542, *p* = 0.590) nor the GPA (estimate = −1.631, *SE* = 4.556, *t* = −0.358, *p* = 0.725) demonstrated significant effects, and the intercept was not significant (estimate = 26.819, *SE* = 14.928, *t* = 1.797, *p* = 0.090).

## 4 Discussion

The main objective of this study was to analyze how different learning environments (face-to-face vs. online) affect students’ sleep, cognitive function, various aspects of sleep patterns, physical activity, and cognitive function. Additionally, this study aimed to assess the influence of students’ GPA and the type of learning environment on each of these dependent variables, while controlling for individual differences among subjects.

A comprehensive analysis of various sleep- and activity-related factors revealed a prevailing trend characterized by a lack of substantial differences between the different learning environments (face-to-face vs. online). Furthermore, no statistically significant variations were observed in the PSQI Score, RVPA, SWMBE, or self-assessment of sleep quality according to the type of learning environment. Additionally, it is worth noting that in several models, the intercepts were significant, emphasizing the importance of baseline levels for these variables.

The absence of a significant effect of teaching environment on sleep suggests that students were able to obtain the necessary amount of sleep during online classes. It is important to emphasize that our university maintained consistent lecture schedules during the day, even when transitioning to online classes with recorded sessions. This strategic decision aimed to mitigate the negative effect of using electronic devices late at night on sleep quality. Although it is evident that the COVID-19 pandemic initially had a detrimental impact on sleep quality, it is worth noting that over the extended period of the pandemic, people gradually regained stability in their daily routines. This may explain why online classes did not have a significant effect on sleep patterns in this study. As individuals adapted to the challenges posed by the pandemic, they were likely to develop strategies to prioritize their sleep while continuing their online education (Walker and Stickgold, 2006; Alhola and Polo-Kantola, 2007).

The absence of pronounced effects on sustained attention and executive function in online classes found in this study can be attributed to a combination of individual differences, adaptation over time, effective teaching practices, and student engagement. This highlights the notion that students’ ability to maintain attention and executive function is influenced by various factors and that the learning environment is just one element in a complex interplay of influences (Ratey and Loehr, 2011; Diamond, 2015). To clarify, the participants in this study were engaged in both theoretical and practical aspects of physical education. The face-to-face learning environment included interactive lectures, group discussions, and practical sessions that involved physical activity. Conversely, the online sessions primarily focused on theoretical knowledge dissemination through multimedia presentations and virtual simulations. This distinction is crucial as it directly influences the engagement levels and physical activity of the students, potentially impacting their sleep patterns and cognitive functions. It is essential to note that these results may be specific to our study group, because students’ learning preferences and experiences can vary significantly. Some individuals thrive in the structured and socially interactive environments of in-person classes, whereas others find online learning more flexible and less distracting. In a meta-analysis conducted by Pashler et al. (2008), weak or no evidence was found to support the effectiveness of learning-style interventions, indicating that individual differences in learning styles may overshadow any overall effect of online learning on sustained attention and executive functioning. It is worth mentioning that the participants in our study had prior experience with online learning environments during the COVID-19 pandemic. This prior experience may have equipped them with better skills in managing distractions and maintaining focus, whereas those new to online learning may have faced initial challenges. A review by Means et al. (2013) acknowledges the potential facilitative role of prior experience in online learning but emphasizes the need for further research to better understand the specific mechanisms at play and their interactions with other factors. In addition, the quality of online course design and delivery plays a significant role in student engagement and attention. Well-designed courses that incorporate interactive activities, provide clear expectations, and offer regular feedback may enhance focus, whereas poorly designed courses could lead to passive learning and increased distraction (Clark and Mayer, 2023).

By contrast, when examining PAL and weekday steps, the present study identified significant effects specifically associated with the face-to-face learning environment, highlighting a notable impact in these areas.

The findings of this study align closely with similar research conducted globally focusing on the realm of online activities. While the number of studies conducted specifically within the domains of physical education and sports departments is relatively limited, there is a broader body of research related to online education across various disciplines. Analyzing these broader studies can shed light on both shared and unique aspects within the field of physical education at universities.

The challenges associated with physical activities are identified in a study conducted by Rodríguez-Larrad et al. (2021) involving 13,754 students from 16 Spanish universities. This study reveals a significant decline in both moderate and vigorous physical activity, with an increase in sedentary behavior affecting more than 50% of the participants. Another study conducted by Wickersham et al. (2021) highlights a noticeable decline in the number of weekly steps taken, reflecting decreased distances traveled during off-campus classes. In the present study, the linear mixed-effects model of mean weekday steps reveals a significant effect of learning environment type. This finding is consistent with previous studies that reported decreases in physical activity among student populations during off-campus classes (Gallo et al., 2020; Savage et al., 2020; Luciano et al., 2021; Mocanu et al., 2021; Rodríguez-Larrad et al., 2021). However, it is worth noting that during the COVID-19 pandemic, Wickersham et al. (2021) find an increase in the number of steps taken from the onset of lockdown and throughout the subsequent easing of restrictions. This likely indicates growing certainty and clarity surrounding the imposed restrictions, coupled with the gradual relaxation of rules, which allowed for more outdoor activities and social interactions (Wickersham et al., 2021). It is important to mention that during our study, no restrictions were imposed when conducting online classes, which may explain the non-significant effect of the type of learning environment on weekends between online and face-to-face classes.

Physical activity may not be the sole factor that affects online classes. Therefore, it is important to consider their impact on cognitive and executive functions, which are known to contribute to academic success. In our study, the linear mixed-effects model identifies a significant difference in PAL between the learning environments. Specifically, notable variations in visual memory and learning abilities were observed when the PAL was used to assess these variables. With the utilization of CANTAB, the outcome measures encompass various factors, including participant errors, number of trials needed to correctly identify patterns, memory scores, and completed stages. The effective storage of information, whether temporary or retrieved, necessitates proficient encoding, manipulation, and retrieval abilities. Consequently, as recommended by Varela-Aldás et al. (2023), the implementation of memory training programs becomes imperative when these deficits are identified. Therefore, it is advisable to integrate memory training programs into online classes.

In our study, when the GPA was included as a covariate, it did not consistently demonstrate a significant influence on various outcomes. Consequently, these findings suggest that academic performance may not be a decisive factor affecting the impact of online classes on sleep, physical activity, or cognitive functioning among physical education students. Further research is required to delve deeper into the intricate relationships among academic performance, online learning, and well-being, considering diverse populations and various influencing factors.

To further enhance the efficacy of online learning environments, educators could integrate structured physical activities and cognitive exercises into the curriculum. Such integration not only counters the sedentary nature of online learning but could also improve cognitive functions, as physical activity is known to have a positive impact on brain health and cognitive performance (Ratey and Loehr, 2011). Specifically, incorporating short physical activity breaks can help maintain students’ physical health and potentially enhance their cognitive engagement and learning outcomes. Moreover, embedding cognitive exercises and interactive learning activities can provide the dual benefits of enhancing cognitive function and mitigating the potential distractions associated with online learning environments (Diamond, 2015). Ultimately, this study underscores the importance of considering various factors, including physical activity and cognitive engagement, in the design and delivery of online learning. As educators continue to navigate the challenges and opportunities presented by online and hybrid learning modalities, incorporating evidence-based strategies that promote students’ physical and cognitive well-being will be crucial to fostering effective and holistic educational experiences (Donnelly and Lambourne, 2011). Furthermore, future research should explore the long-term effects of different learning environments, considering both extended study periods and follow-up assessments, as highlighted by Vaalayi et al. (2023) and Rassolnia and Nobari (2024). The study by Vaalayi et al. (2023) demonstrated that low-intensity aerobic exercise can attenuate the negative effects of partial sleep deprivation on cognitive performance, suggesting that integrating physical activities in online learning could mitigate some of the cognitive challenges posed by reduced physical activity. Similarly, Rassolnia and Nobari (2024) found that socio-economic status and physical activity significantly influence psychological well-being and sleep quality among college students, highlighting the importance of promoting physical activity to improve overall student health and academic performance.

## 5 Conclusion

A comprehensive analysis of various sleep- and activity-related variables revealed a pattern of mostly non-significant differences between the learning environments (face-to-face vs. online). Specifically, for the PSQI Score, RVPA, SWMBE, and the self-rating scale of sleep quality, no significant variations were found in relation to the type of learning environment. However, in the case of PAL and weekday steps, significant effects were observed in the face-to-face learning environment, indicating a significant impact on these areas. Additionally, the intercepts in several models were significant, highlighting the importance of the baseline levels of these variables. The GPA, included as a covariate, did not demonstrate a consistently significant influence on the outcomes. These findings suggest that while the learning environment type notably influences certain activity metrics, such as PAL and weekday steps, its overall impact on sleep quality and other activity metrics is generally limited. To deepen our understanding of these phenomena, future research should explore the long-term effects of online learning on critical health and cognitive domains. Investigating potential interventions to enhance physical activity and cognitive function in online learning environments is crucial for developing more holistic educational models. Additionally, examining the impacts of various learning modalities on students’ well-being and academic performance can offer valuable insights for educators and policymakers aiming to optimize educational practices in a digital landscape.

While our study provides significant insights into the impacts of learning environments on physical education students, it is imperative to consider the broader applicability of these findings. Further investigation is needed to determine how these results translate to students in other academic disciplines or educational settings, which would enhance the generalizability and relevance of our conclusions. Moreover, the focus of this study on male students raises questions about the generalizability of our findings across genders. Future research should include a more diverse participant pool, encompassing both male and female students, to explore potential gender differences in how learning environments affect sleep, physical activity, and cognitive function. Such inclusive research efforts will contribute to a more comprehensive understanding of the educational landscape, supporting the development of strategies that cater to a wider array of student needs and preferences.

## Data availability statement

The raw data supporting the conclusions of this article will be made available by the authors, without undue reservation.

## Ethics statement

The studies involving humans were approved by Qatar University’s Institutional Review Board. The studies were conducted in accordance with the local legislation and institutional requirements. The participants provided their written informed consent to participate in this study.

## Author contributions

MH: Writing – review & editing, Writing – original draft, Visualization, Validation, Supervision, Software, Resources, Project administration, Methodology, Investigation, Funding acquisition, Formal analysis, Data curation, Conceptualization. ZA: Writing – review & editing, Writing – original draft, Visualization, Validation, Supervision, Software, Resources, Project administration, Methodology, Investigation, Data curation, Conceptualization. A-SA-S: Writing – review & editing, Writing – original draft, Visualization, Validation, Supervision, Methodology, Investigation, Funding acquisition, Formal analysis, Data curation, Conceptualization.
